# Mycosynthesis of silver nanoparticles and their characterization

**DOI:** 10.1016/j.mex.2017.12.003

**Published:** 2018-01-17

**Authors:** M. Madakka, N. Jayaraju, N. Rajesh

**Affiliations:** a Dept., of Biotechnology and Bioinformatics, Yogi Vemana University, Kadapa, 516 003, AP, India; b Dept., of Geology, Yogi Vemana University, Kadapa, 516 003, AP, India

**Keywords:** AgNPs, Extracellular biosynthesis, *Aspergillus niger*, Fusarium semitectum, Antimicrobial effect

## Abstract

Mycosynthesis deal with an energy-saving and eco-friendly process intended for extracellular synthesis of AgNPs, by means of cell-free filtrates of fungi *Aspergillus niger* and *Fusarium semitectum* as reducing agents. Optimization of different parameters during biosynthetic process demonstrated diverse property on production rate, the size, distribution, yield of biosynthesized AgNPs. SEM micrographs showed polydisperse spherical and ellipsoid nanoparticles (SIZE). AgNPs exhibits potential antimicrobial effect than Ag^+^ not in favor of *E. coli, Staphylococcus aureus*, and *Pseudomonas aeruginosa*. These results demonstrate that mycosynthesis of AGNPs is a cost effective and eco-friendly method, resulting in particles with antibacterial properties that are efficient as an antimicrobial agent.

• Characterization of Silver nano particle is widely applauded domain at present.
• Mycosynthesis of AgNPs as reducing agents and exhibits potential antimicrobial effect.
• Results of mycosynthesis of AgNPs is cost effective and ecofriendly.

## Graphical abstract

**Figure:**
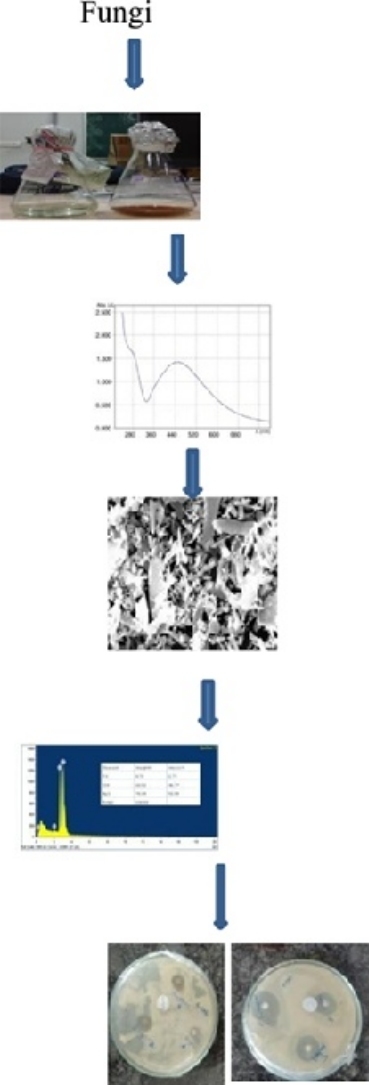

## Method details

Nano-biotechnology deals with the production as well as stabilization of a variety of nanoparticles. At present, here is an urgent necessary to increase biodegradable process for the production of AgNPs. The center of attention for this production has modify from physical and chemical process towards ‘green’ chemistry and bioprocesses Vigneshwaran et al., 2007. A number of pattern of AgNPs development through organisms include magnetotactic bacteria synthesizes magnetite nanoparticles Lowenstam et al., 1981 and S-layer bacteria producing calcium carbonate and gypsum layers [1]. Biomacromolecules and Proteins manage the nucleation and development of these inorganic structures [[2], [3]]. AgNPs are an efficient antimicrobial mediator acts against a variety of harmful pathogens, as well as a variety of chemical and biochemical methods being surveyeed for its production [4]. A variety of microbes be recognized to reduce metal ions to metals [[5], [6], [7]]. The resistance conferred through microbes in the direction of Ag^+^ is determined by the ‘sil’ gene in plasmids [8]. *Fusarium oxysporum* strains has been recognized to reduce Ag^+^ ions to a nitrate-dependent reductase and a shuttle quinone extracellular method [9]. Active metal conversion procedure requires viable microorganisms, which enzymatically catalyze the modification of the metal. The microorganisms take part in a major role in providing a large amount of nucleation centers and establish circumstances for obtaining extremely disperse nanoparticle systems. They slow down, or entirely prevent, aggregation through immobilizing the particles and providing a viscous medium [10]. In the remediation of toxic metals, microbes, such as fungi and bacteria, are engaged. Hence microbes have newly been documented as possible eco-friendly nanofactories [11]. Many investigators have described the synthesis of AgNPs [[12], [13], [14], [15], [16], [17]] and gold [18] nanoparticles through microbes. This current study describes the extracellular production of stable AgNPs using the fungi *Fusarium semitectum* and *Aspergill us niger*. Use of these fungal strains for nanoparticle biosynthesis is technologically attractive, reasonable, and commercially feasible.

## Materials and methods

### Preparation

Analytical grade chemicals were used in the current study. Silver nitrate (Ag NO3) be procure from Sigma-Aldrich Chemicals (USA). Fungal and bacterial growth medium were procured from Oxoid Company (UK). The three test bacteria used for the antimicrobial study were procured from the Microbial Type Culture Collection (MTCC) (India).

### Extracelluar production of AgNPs

Fungal strains *Aspergillus niger* was grown in czapadox broth, where as *Fusarium semitectum* was grown in glucose nutrient broth medium (GNB), with adjument of pH 7.0. The two fungal strains were kept in the rotary shaker at 120 rpm at 280C. Centrifuge the two mycelia broth separately at 6000 rpm and separate the mycelium after 3 days of incubation, followed by washing with deionized water thrice. Suspend the two strains of washed mycelium separately with deionized water and kept for incubation for 4 days in shaking incubator for at 180 rpm. The fungal supernatant obtained after removal of mycelium from two strains was confront with 100 mL of 1 mM AgNO3 solution (prepared in deionized water) and kept in dark conditions at 280C in rotary shaker at 120 rpm. All together, a positive control was maintained by incubating the fungal supernatant through deionized water was maintained under the similar conditions. Purification of AgNPs was done by centrifugation. The silver colloids were washed with deionized water at least three times under an N2 stream, to remove excess silver ions followed by freeze-drying to obtain a dry powder of the nanosize silver. The freeze dried silver nanoparticle was resuspended in deionized water to carry out further interaction with microbes and characterisation of nanoparticles [9].

### Characterization of nanoparticles

The biosynthesis of AgNPs by *Aspergillus niger* and *Fusarium semitectum* was visually monitored by UV–vis spectrophotometer 119 (Systronics) analysis by monitoring absorption spectra between 250 nm to 750 nm of the reaction mixtures of AgNO3 and aqueous extract by withdrawing 5 mL aliquots. Freeze dry the reaction mixture containing AgNPs for further characterization. Vacuum freeze dryer (Ref-Vac Consultancy, India) of a laboratory-scale which contains of a drying chamber with stainless steel, −35° C maintaining condenser, and for freezing a vacuum pump (Crompton Greaves, India). The sample was frozen in an external deep freezer (Blue Star, India) at −280C for 6 h. The freeze dryer was started, and allow the condenser to reach at least −35° C. Sample was positioned in the aeration chamber and the vacuum pump, heater were started. The temperature of the sample was set at 25° C and the pressure inside the chamber was set to 30 Pa, respectively. For scanning electron microscopy (SEM) investigation of the freeze-dried sample was used by mounting nanoparticles on sample stubs with double-sided adhesive tape and coating with platinum in a sputter coater and examining under a JEOL 63861 SEM (Japan) at 10 kV. The freeze-dried reaction mixture embedded with the AgNPs was used for X-ray diffraction (XRD) analysis. XRD patterns be recorded on X’Pert Pro, PANalytical (USA), working at 40 kV and a current of 30 mA with Cu Ka radiation (l = 1.54A°). The diffracted intensities were recorded from 58 to 1208 2 u angles.

### Antimicrobial activity assay

Kirby- Bauer disc diffusion method was employed for the antimicrobial activity of biosynthesized AgNPs against *Escherichia coli (*ATCC-8739) and *Pseudomonas aeruginosa* (ATCC-15442); and *Staphylococcus aureus* (ATCC-6538). Pre-cultivate the bacteria at 37 °C 24 h on nutrient agar slant by pricking a single colony of microbial strains. To prepare the inoculum of each strain, wash and suspend the cultures from slants in 0.9% Nacl with a density equivalent to the 0.5 McFarland standards (1.5 × 108 CFU/mL). Inoculate the adjusted inoculum on Muller-Hinton agar plates. Add 20 μL of different concentrations of biosynthesized AgNPs (50, 100 and 200 μg/ml) on to sterilized paper disks (6 mm diameter) and placed on inoculated plates. For comparison the cell-free filtrate of *Aspergillus niger* and *Fusarium semitectum* and AgNO3 (100 μg/ml) was also tested. Incubate all of the plates were incubated at 37 °C and 28 °C for 24 h and 48 h. Diameter of the inhibition zone around each disc was measured and represented as the antibacterial activity for each of the test samples after incubation. The assays were implemented three times [19].

## Results and discussion

### AgNPs biosynthesis

**Fig. 1:**
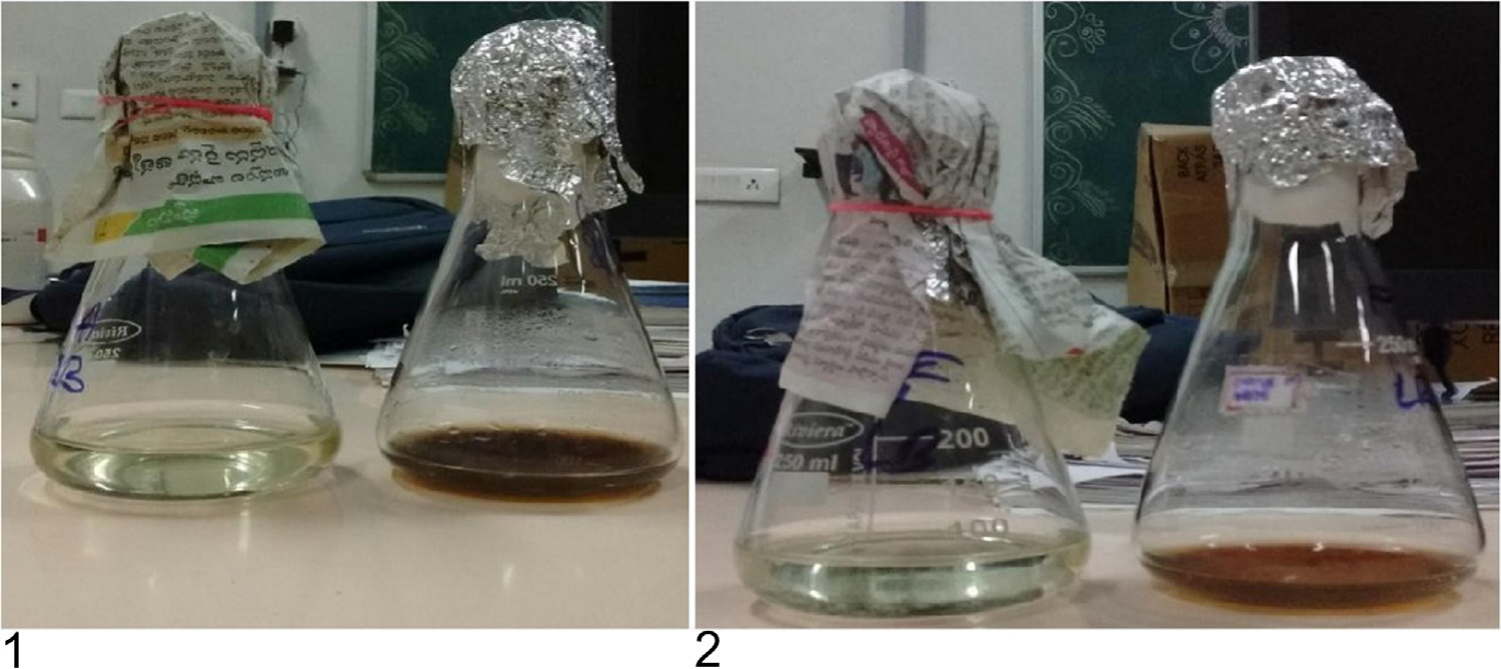
(1) & (2) Aqueous extract of silver nitrate solution(control) and Fungal aqueous extract with silver nitrate (Formation of nanoparticles,dark brown colour) of Fusarium and Aspergillus niger.

In this work, the fungi *Fusarium semitectum* and *Aspergillus niger* were used for the production of stable AgNPs and were scrutinized visually by turning the fungal aqueous filtrate incubate with AgNO3 solution into dark brown due to the deposition of AgNPs due to the surface plasma resonance. (Fig. 1). No color change in color was observed in the control was observed. According to Bansal et al. [20] the invitro development of a stable Ag^+^ hydrosol AgNPs with diameter of 10–25 nm which was stable by a capping by using α-NADPH-dependent nitrate reductase and phytochelatin. The formation of AgNPs was indicated by the, color change in the solution. After 1 day of incubation, the AgNPs were well discrete in the solution without any aggregation, with varying the color concentration of the cell filtrate with AgNO3. This is due to the surface Plasmon resonances (SPR) effect and reduction of AgNO3 [20] AgNPs produce a brown solution in water.

### UV- visible analysis

**Fig. 2:**
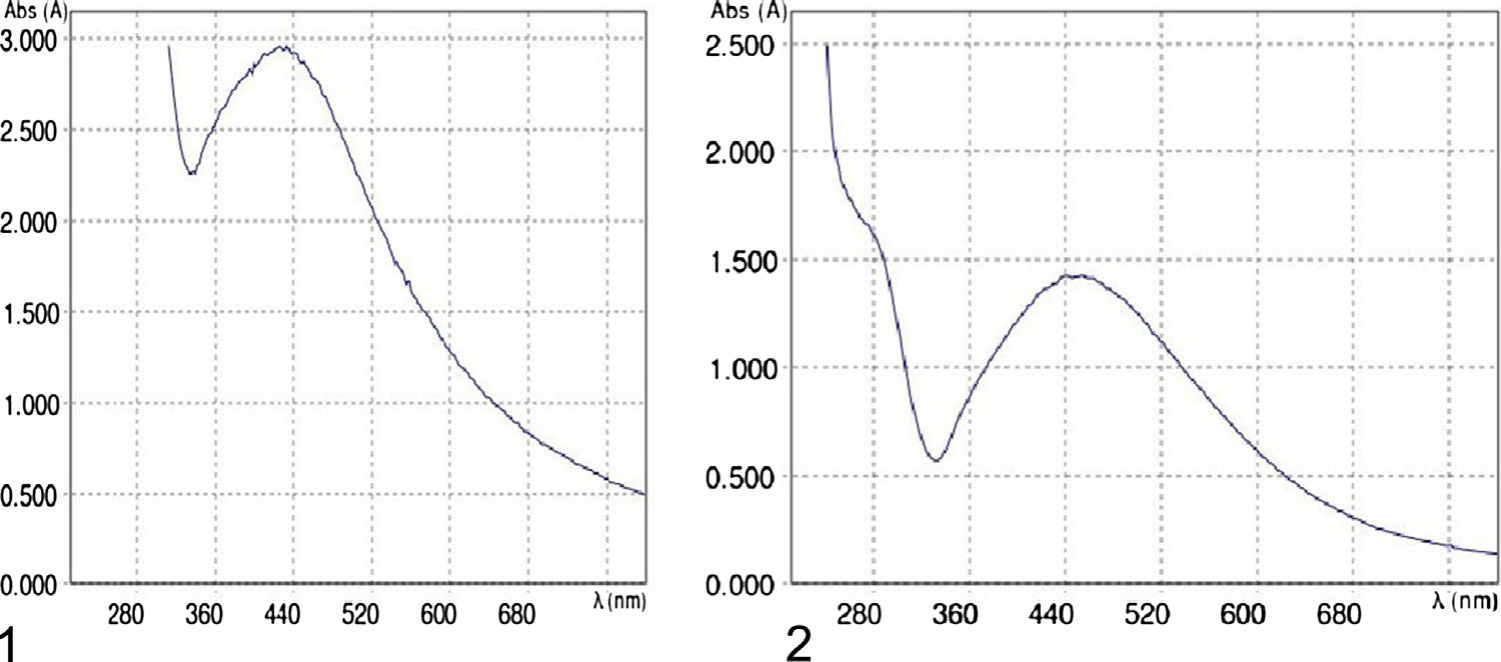
(1) & (2): UV spectrum of reaction mixture of silver nitrate and aqueous filtrate of *Fusarium semitectum* and *Aspergillus niger* with peaks at 420 nm and 450 nm. (1) The peak was observed at 420 nm. (2) The peak was observed at 450 nm.

UV–vis spectral study is to mointer the arrangement and constancy of the reduced AgNPs in colloidal solution. (Fig. 2). Silver surface Plasmon resonance spectral band occurred at 420 nm of *Fusarium semitectum* based nanoparticles and 450 nm of *Aspergillus niger* based nanoparticles at diverse time intervals of reactions. No further increase in intensity was recorded after 3 days of incubation, indicating the precursor silver ions were reduced. The presence of AgNPs was indicated by UV–vis spectra of the cell deposit with AgNO3 showed a strong extensive peak at 440 nm which is surface Plasmon resonances (SPR band). These above results were similar with the information of Verma et al. [21].

### Scanning electron microscopy (SEM)

**Fig. 3:**
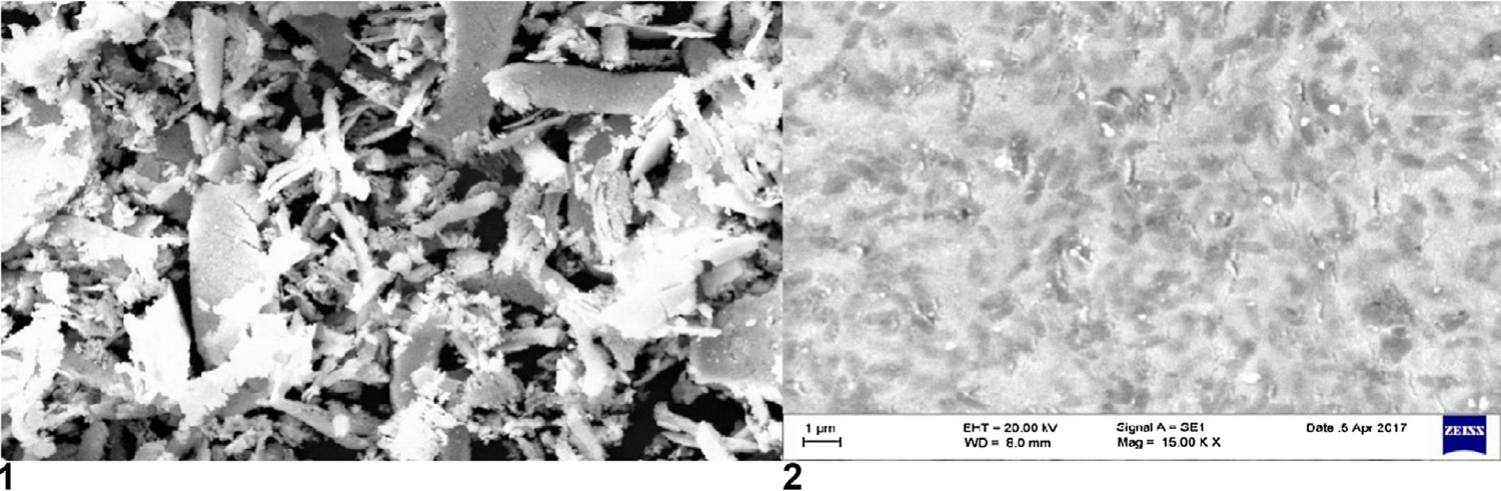
(1) & (2): Scanning electron micrograph of the freeze-dried silver nanoparticles synthesized after silver nitrate of reaction mixture of silver nitrate and aqueous filtrate of Fusarium and Aspergillus niger.

Scanning Electron Microscopy (SEM) investigation image provides additional insight into the shape and dimension of the nanoparticles. It is evident from the figure that the bio-synthesized silver nanoparticles are in small and spherical in shape which indicates the surface deposited silver nanoparticles Fig. 3(1) & (2). Monodispersity, shape, size of particles were the highly dependent on function of AgNPs. These above results were similar with the information Pal et al. Pal et al. (2006).

### Energy dispersive spectroscopy (EDS)

**Fig. 4:**
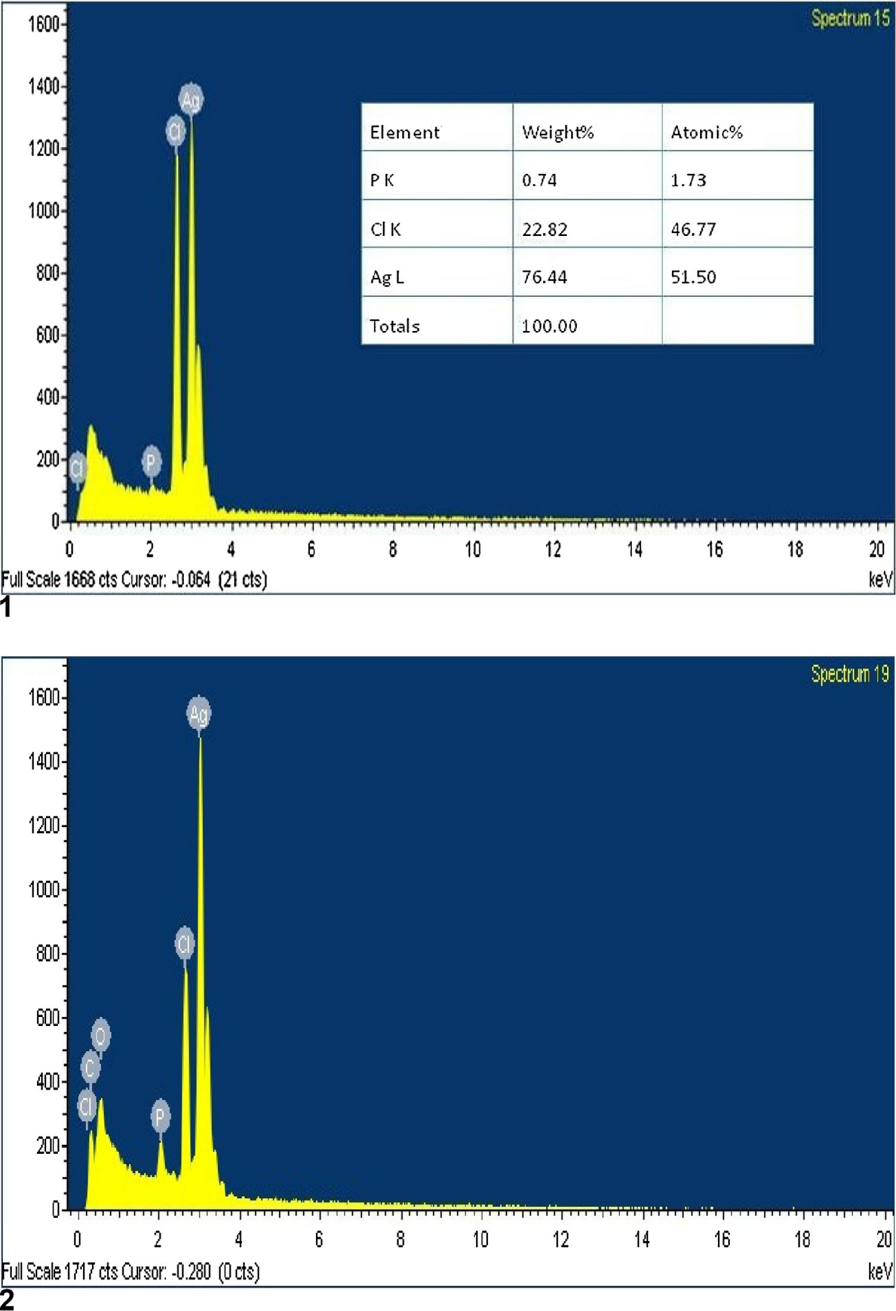
(1) & (2): Energy dispersive spectroscopy Scanning electron micrograph of the silver nanoparticles synthesized after silver nitrate of reaction mixture of silver nitrate and aqueous filtrate of *Fusarium semitectum and Aspergillus niger.* (1) EDS of silver nanoparticles of Fusarium semitectum nanoparticles. (2) EDS of Silver nanoparticles of Aspergillus niger.

Samples for energy dispersive spectroscopy (EDS) were arranged on a cu^+^ substrate through drop covering of AgNPs of nanoparticles produced by the two different organisms. JEOL (JSM-6380 LA) SEM was used for the approval of elemental study of single particles. Based on this the weight of silver was 76.44% of *Fusarium semitectum* nanoparticles (Fig. 4a) and 74.17% of *Aspergillus niger* nanoparticles (Fig. 4b).

### Antimicrobial properties of silver nanoparticles of *Fusarium semitectum* and *Aspergillus niger*

The diameters of zone of inhibition were equal for all the tested bacteria when compared with the control (Table 1) which indicates that the AgNPs might be consider as tremendous broad spectrum antibacterial agents against the pathogens. AgNPs show considerable antimicrobial activity, they can have the potential to be broadly used in medical applications. Antibacterial activity of AgNPs has been confirmed in several investigations. In the present study, AgNPs showed high-quality antibacterial activity against all the tested bacteria due to the differences in bacterial cell walls. Li and coworkers [22] proposed the mechanism of AgNPs. include the following: The accurate method of antibacterial effect of AgNPs on microorganisms is to be evaluated, but it can be associated to the mechanism of Ag+ ions action against bacteria strains such as *E.coli, Staphylococci* and *Pseudomonas* where by the rate of enhance of AgNPs from the aqueous solution ultimately causes dispersion of protein and enzymes in the cell. As a substitute, here are following potential antimicrobial mechanisms-

**Table 1:** Zone of inhibition of *Fusarium semitectum.*

S.No	Name of the organism	Zone of inhibition					
		Fusarium semitectum			Streptomycin		
		1 μg/ml	2 μg/ml	5 μg/ml	1 μg/ml	2 μg/ml	5 μg/ml
1.	E.coli	0.3	0.8	1.0	2.0	1.0	1.0
2.	Staphylococci	0.5	0.7	2.5	–	1.4	1.7
3.	Pseudomonas	0.3	0.5	0.8	–	1.0	2.0

(a) Bacterial development and propagation are negatively reserved, consequential change in the cell wall which in turn is not capable to shield the internal of the cell by adhesion of ultra-small sized AgNPs onto the cell wall of bacteria (b) The diffusion of AgNPs into the bacterial cell leads to DNA break, or death of the cell, by varying its regular performance of bacterial nuclear content; and (c) The permanent disruption of bacterial cell wall was caused by the interaction Ag+ ions with sulfur containing proteins present in the bacterial cell wall. The main antibacterial mechanism in evaluating the antibacterial effect of the proposed mechanism (Table 2).

**Table 2:** Zone of inhibition of *Aspergillus niger.*

S.No	Name of the pathogen	Zone of inhibition(mm)					
		Fusarium semitectum			Streptomycin		
		1 μg/ml	2 μg/ml	5 μg/ml	1 μg/ml	2 μg/ml	5 μg/ml
1.	E.coli	0.5	0.7	1.0	1.0	1.0	2.0
2.	Staphylococci	0.5	1.0	2.0	–	1.4	1.7
3.	Pseudomonas	0.3	0.7	1.0	–	1.0	2.0

**Fig. 5:**
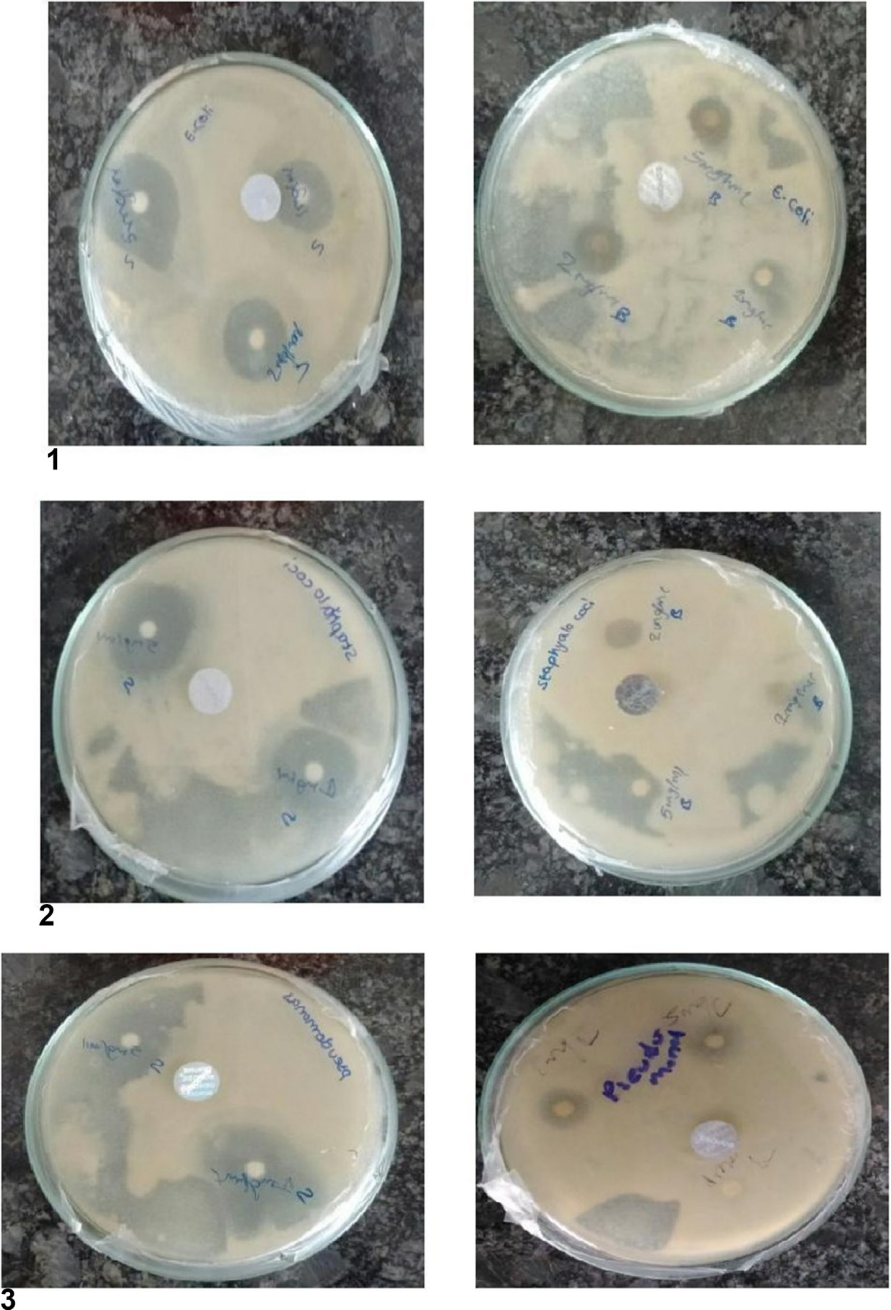
Antimicrobial activity of the silver nanoparticle synthesized after silver nitrate of reaction mixture of silver nitrate and filtrate of Fusarium semitectum and *Aspergillus niger.* (1) Antimicrobial assay of silver nanoparticle by *E.coli*. (2) Antimicrobial assay of silver nanoparticle by *Staphylococci*. (3) Antimicrobial assay of silver nanoparticle by *Pseudomonas*.

**Fig. 6:**
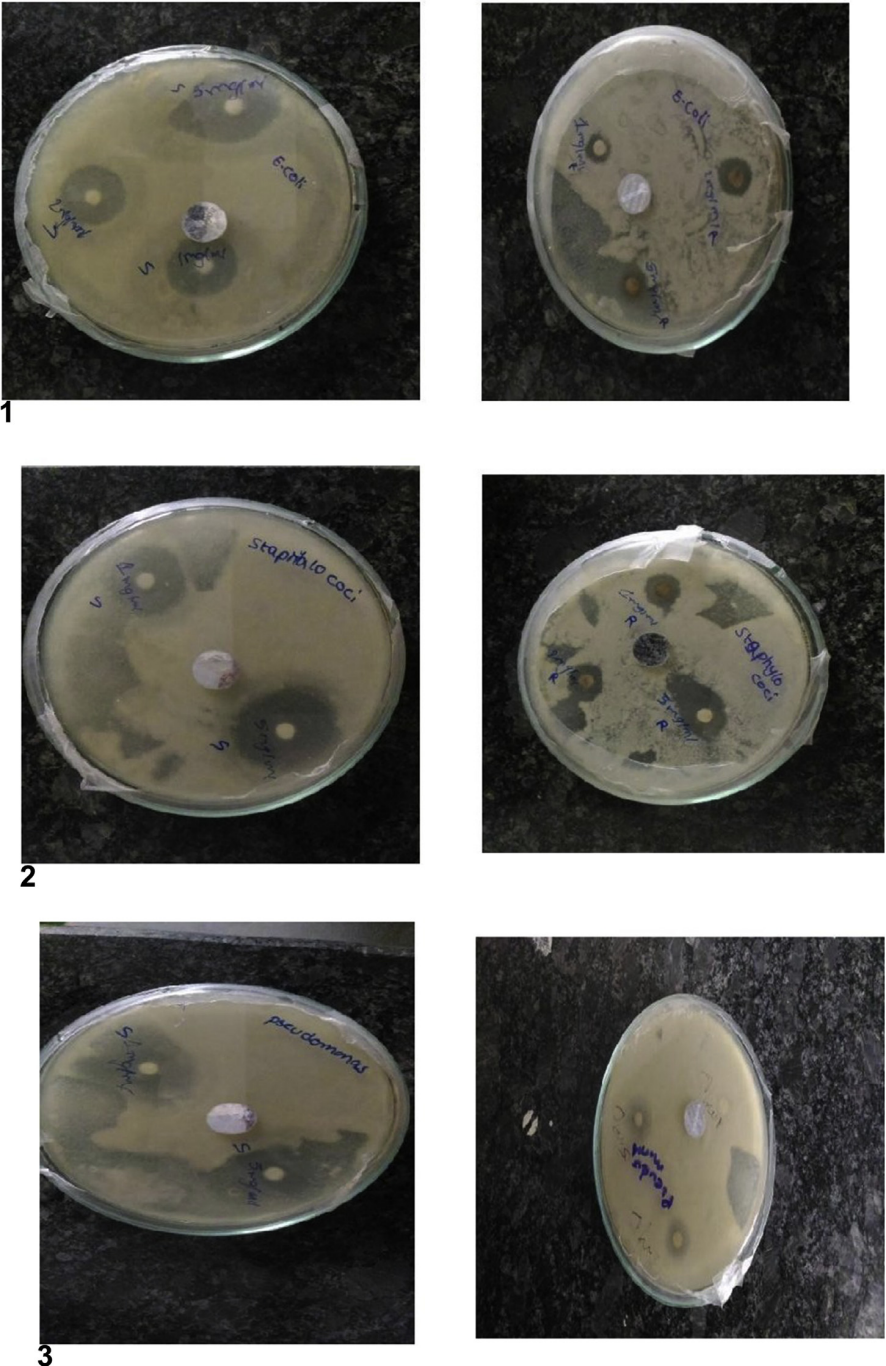
Antimicrobial activity of the silver nanoparticle synthesized after silver nitrate of reaction mixture of silver nitrate and filtrate of Fusarium semitectum and *Aspergillus niger.* (1) Antimicrobial assay of silver nanoparticle by *E coli*. (2) Antimicrobial assay of silver nanoparticle *Staphylococci*. (3) Antimicrobial assay of silver nanoparticle by *Pseudomonas.*

The AgNPs antimicrobial activity depends on shape, dimension, and the exterior charge of the particles. AgNPs have superior antibacterial effect as they can effortlessly go through into the nucleus of microbe owing to the organization of their microbial cellwall, particularly in gram^−ve^ bacteria, suppressing the DNA and enzymes and principal to cellular fatality. They can also seize better surface area for stronger bactericidal communications [23]. We can also use streptomycin because it can act against bacteria such as *E.coli, Staphylococci* and *Pseudomonas* (Fig. 5, Fig. 6).

In the current study, AgNPs showed high-quality antibacterial activity against all the tested bacteria, due to the differences in bacterial cell walls and concentration and size of the AgNPs as well as the bacterial initial concentration [24].

### Conclusion

Mycosynthesis of nanoparticles is relatively new in the field of research involving microorganisms. Among other organisms fungi makes a suitable option for the production of metallic nanoparticles because they secrete large amount of proteins, thus increasing productivity, and their easy usage in the laboratory. The present study has reported the biological procedure for the production of extracellular silver nanoparticles using *Fusarium semitectum* and *Aspergillus niger.* Further characterization was made by UV–vis spectroscopy which showed maximum absorption at 420 nm and 450 nm while the Scanning Electron Microscope (SEM) discovered the structure of spherical nanoparticles with no agglomeration. Biosynthesized silver nanoparticles have more potent antibacterial activity against *E.Coli, Staphylococci* and *Pseudomonas* of spherical nanoparticles than antibiotics. Thus, results conclude that isolated *Fusarium semitectum* and *Aspergillus niger* are prominent producers of silver nanoparticles a have strong antimicrobial activity against pathogenic bacteria. Currently there is an increasing demand to prepare AgNPs for different medical and industrial purposes because biosynthesized silver nanoparticles have a broad range of applications.
